# Cochlear Implant Programming: A Global Survey on the State of the Art

**DOI:** 10.1155/2014/501738

**Published:** 2014-02-04

**Authors:** Bart Vaerenberg, Cas Smits, Geert De Ceulaer, Elie Zir, Sally Harman, N. Jaspers, Y. Tam, Margaret Dillon, Thomas Wesarg, D. Martin-Bonniot, L. Gärtner, Sebastian Cozma, Julie Kosaner, Sandra Prentiss, P. Sasidharan, Jeroen J. Briaire, Jane Bradley, J. Debruyne, R. Hollow, Rajesh Patadia, Lucas Mens, K. Veekmans, R. Greisiger, E. Harboun-Cohen, Stéphanie Borel, Dayse Tavora-Vieira, Patrizia Mancini, Helen Cullington, Amy Han-Chi Ng, Adam Walkowiak, William H. Shapiro, Paul J. Govaerts

**Affiliations:** ^1^The Eargroup, Herentalsebaan 75, 2100 Antwerp-Deurne, Belgium; ^2^Laboratory of Biomedical Physics, University of Antwerp, Antwerp-Deurne, Belgium; ^3^VU University Medical Center, 1081 HZ Amsterdam, The Netherlands; ^4^Hôpital Sacré Cœur, Beirut B.P.116, Lebanon; ^5^Yorkshire CI Service, Bradford BD9 5HU, UK; ^6^University Hospital Saint-Luc, 1200 Brussels, Belgium; ^7^Emmeline Centre, Cambridge CB2 0QQ, UK; ^8^University of North Carolina at Chapel Hill, Chapel Hill, NC 27514, USA; ^9^University Medical Center Freiburg, 79106 Freiburg, Germany; ^10^CHU de Grenoble, 38701 Grenoble, France; ^11^Medizinische Hochschule, 30625 Hannover, Germany; ^12^University of Medicine and Pharmacy Grigore T. Popa, 95060 Iasi, Romania; ^13^Meders Hearing and Speech Center, Istanbul, Turkey; ^14^University of Kansas Medical Center, Kansas City, MO 66160, USA; ^15^Dr Manoj's ENT Superspeciality Institute & Research Centre, Calicut, Kerala 673005, India; ^16^LUMC, 2300 RC Leiden, The Netherlands; ^17^Royal National Throat, Nose and Ear Hospital, London WC1X 8EE, UK; ^18^University Hospital, 6202 AZ Maastricht, The Netherlands; ^19^Royal Victorian Eye and Ear Hospital, Melbourne, VIC 3002, Australia; ^20^PD Hinduja Hospital National and MRC, Mumbai 400016, India; ^21^UMC St. Radboud, 6525 GA Nijmegen, The Netherlands; ^22^Nottingham Auditory Implant Programme, Nottingham NG1 5DU, UK; ^23^University Hospital, 27 Oslo, Norway; ^24^Hôpital Rothschild, 75012 Paris, France; ^25^Hôpital Beaujon, Paris, France; ^26^Medical Audiology Services, Perth, WA 6005, Australia; ^27^University Sapienza, 185 Rome, Italy; ^28^South of England CI Centre, Southampton SO17 1BJ, UK; ^29^Sunnybrook, Toronto, ON, Canada M4N 3M5; ^30^World Hearing Center, 02-042 Warsaw, Poland; ^31^NYU Langone Medical Center, New York, NY 10016, USA

## Abstract

The programming of CIs is essential for good performance. However, no Good Clinical Practice guidelines exist. This paper reports on the results of an inventory of the current practice worldwide. A questionnaire was distributed to 47 CI centers. They follow 47600 recipients in 17 countries and 5 continents. The results were discussed during a debate. Sixty-two percent of the results were verified through individual interviews during the following months. Most centers (72%) participated in a cross-sectional study logging 5 consecutive fitting sessions in 5 different recipients. Data indicate that general practice starts with a single switch-on session, followed by three monthly sessions, three quarterly sessions, and then annual sessions, all containing one hour of programming and testing. The main focus lies on setting maximum and, to a lesser extent, minimum current levels per electrode. These levels are often determined on a few electrodes and then extrapolated. They are mainly based on subjective loudness perception by the CI user and, to a lesser extent, on pure tone and speech audiometry. Objective measures play a small role as indication of the global MAP profile. Other MAP parameters are rarely modified. Measurable targets are only defined for pure tone audiometry. Huge variation exists between centers on all aspects of the fitting practice.

## 1. Introduction

Cochlear implants (CI) processors must be appropriately programmed and customized for the recipient [1, 2]. The aim of this is to set a number of parameters to ensure that the electrical pattern generated by the device in response to sound yields optimal speech intelligibility. Several electrical parameters are available and all their values together is commonly called the MAP. Finding and programming the optimal values for a recipient is commonly called the act of fitting. It is achieved using proprietary software and a hardware interface connected to the processor and depends on behavioral responses from the CI recipient.

After the initial switch-on or activation of the processor, several fitting sessions are normally required [3]. Most of the MAP adjustments take place over these first few months, until levels remain relatively stable [3–5]. Following stabilization of electrical dynamic range, fitting sessions are usually limited to periodical checks, typically annually, as long as progress remains satisfactory.

Training in fitting is usually provided primarily by the CI manufacturers, and, although there are guidelines and recommendations, no standardized methodology exists. There are no agreed standards or targets for what should be adjusted or the outcomes expected; as a consequence the MAP a recipient receives could be very different depending on the center visited and the individual heuristics of the audiologist responsible. Most implant teams have an expert opinion of what the expected level of performance for an individual recipient should be and more detailed adjustments are made to the MAP if this target is not reached.

This paper attempts to describe the current state of the art by providing a comprehensive inventory of the fitting strategies in a substantial number of CI centers worldwide. It is beyond the scope of this paper to explain the meaning of all possible MAP parameters or settings. For this information, the reader is referred to the companies' user manuals and to existing comprehensive overviews [2, 6].

## 2. Material and Methods

In preparation for an international debate which was organized in Antwerp, Belgium, in October 2012, a questionnaire was distributed to 47 CI centers worldwide. All questionnaires were returned. All responses were analyzed and the data were discussed during the two-day debate. After this debate all centers were invited to a remote interview (telephone or Skype) to clarify and correct the answers where needed. In addition, the participating centers were invited to log one single fitting session in 5 consecutive recipients of one same CI brand in the months of September-October 2012. This yielded a prospective cross-sectional snapshot of the actual fitting procedure which served as verification for the questionnaire statistics.

The questionnaire is available online (see Supplementary Material available online at http://dx.doi.org/10.1155/2014/501738). Briefly the questions focused on the following topics:

- number of implant recipients being followed and the annual increase,
- brands of implants being implanted and fitted,
- MAP parameters being modified from default at switch-on and during the followup,
- assessments undertaken (subjective, objective, and psychoacoustic) and used to steer the MAP modifications,
- well defined targets used.

The cross-sectional log files contained for each subject the actual values of the different MAP parameters and whether they had been modified during the session under study. In addition, they also contained the information on whether or not objective or psychoacoustic measures were executed during the session.

We summarized all answers either numerically (counts, percentages) or categorically (e.g., the categories: never, exceptionally, sometimes, regularly, and always). In addition, all supplementary information, nuances, and specifications were recorded when relevant.

Descriptive statistics were used and the results are presented graphically by means of histograms or box and whisker plots. Distributions are described by medians, quartile ranges (QR: between 25th and 75th percentile), extremes (minimum and maximum), and outliers.

The term Cochlear is used for the Nucleus device (Cochlear Corporation, Sydney Australia), Med-El for the Med-El device (Med-El, Innsbruck Austria), AB for the Advanced Bionics device (Advanced Bionics Corporation, Valencia, California), and Neurelec for the Digisonic device (Neurelec, Vallauris, France). Throughout the text, the term minimum level is used for the T, THR, T, or MIN parameters of Cochlear, Med-El, AB, and Neurelec, respectively. The term maximum level is used for the C, MCL, M, or MAX parameters. In this paper the term eCAP (electrically evoked compound action potential) is interchangeable with eCAP threshold measurements and refers to (t)NRT, (t)ART, and (t)NRI for Cochlear, Med-El, and AB, respectively.

## 3. Results

### 3.1. Participating Centers

Forty-seven centers from 17 different countries (Australia, Belgium, Canada, France, Germany, India, Italy, Lebanon, Morocco, Norway, Poland, Romania, Spain, The Netherlands, Turkey, United Kingdom, and USA) and 5 different continents (Europe with 60% of centers, North-America 11%, Asia 4%, Australia 4%, and Africa 2%) filled out the paper survey (see full list at the end of the paper). All together they were following 47600 CI users with an annual increase of 4800. Twenty-nine centers had a representative being interviewed. They were following 37000 CI recipients with an annual increase of 3700. This means that the responses of 62% of the participating centers were double-checked covering 78% of the CI recipients being followed. The cross-sectional snapshot yielded data from 255 fitting sessions of 34 centers.

The participating centers have an average experience of 21 years (median startup in 1991; QR: 1987–2000) and a median number of 625 implants (QR: 338–1300) with 62 new implants last year (QR 50–123).

On average each center provides three CI brands (Cochlear, Med-El, and AB); 10.5% provide only 1 brand; 10.5% provide 2 brands; 55% provide 3 brands, and 24% provide 4 brands. The predominant device is Cochlear in 43% of the centers, Med-El in 29%, AB in 25%, and Neurelec in 4%. For all three major brands we received responses from at least 26 centers of which at least 15 were interviewed afterwards. Only Neurelec was underrepresented, with 4 centers responding on paper of which 3 were interviewed. For the cross-sectional verification, at least 14 centers returned the log files of 5 consecutive CI users for each of the major brands. For Neurelec 7 centers returned the log files.

79.5% of centers in the study provide implants to both children and adults, 17% to adults only, and 3.5% to children only.

### 3.2. Switch-On Procedures

On average, the CI processor is switched on after 28 days (QR: 21–30) with some centers starting after 2 weeks (Perth, Melbourne, and Chapel Hill) while one center only hooks up the processor after 6 weeks (Cambridge).

All centers (100%) start with impedance measurements and if short or open most of them (60%) deactivate the corresponding electrodes immediately. Two centers (Brussels, Freiburg since 2013) systematically execute pure tone audiometry prior to switch-on to assess possible residual hearing, while another centre (Hannover) does this during the switch-on week (see further).

Most, if not all, centers' focus goes to the setting of the minimum and the maximum current level of the electrodes. Med-El has a default THR level of 0 and 70% of centers do not change this. AB recommends setting the T level at 10% of the M level and 22% of centers do so. A majority of centers (55%) only determine either the minimum (31%) or the maximum (24%) level and make the other level depend on the first one. Forty-five percent of centers determine both the minimum and the maximum level behaviorally.

#### 3.2.1. Determine Minimum Level Alone

If only the minimum level is determined, this is either done behaviorally (56%) or by means of intraoperative or postoperative eCAP thresholds (44%). The eCAP measures are mostly followed by behavioral verification and adjustment if necessary. Most centers (78%) only determine the minimum levels on a few electrodes and interpolate the values obtained to the other electrodes. Maximum levels are then positioned at one or more intervals above the minimum levels and most centers (67%) perform some form of loudness balancing before switching on the microphone. One centre (Leiden) uses a preset profile of maximum levels which is positioned above the determined minimum levels.

#### 3.2.2. Determine Maximum Level Alone

Determining only the maximum level is restricted to Med-El and AB implants where the minimum level is then set at 0 or 10% of the maximum level. The maximum level is either determined behaviorally (71%) or by means of objective measures (eCAP in 29%, which is combined with or replaced by ESRT (electrically evoked stapedius reflex thresholds) in 14%). If objective measures are used, behavioral verification is done by half of the centers. Interpolation is used in only a minority of centers (29%) and so is loudness balancing (43%).

#### 3.2.3. Determine Both Minimum and Maximum Level

Many centers determine both the minimum and the maximum levels and they all do this behaviorally. Only 15% of these combine this with eCAP measures. One center (Antwerp) has a particular way of using preset MAPs with minimum and maximum levels based on statistical analysis of MAPs which have provided good results in other recipients [7, 8]. These preset MAPs are given without any prior behavioral or other evaluation. Most centers (69%) measure the levels on a number of electrodes and interpolate the levels on the other electrodes. In some cases this can be as few as 3 electrodes (Southampton, Iasi), the results of which are then used to shift a preset profile towards the measured levels. Most centers perform some kind of loudness balancing (62%).

In general, if the maximum levels are measured rather than being derived from the minimum levels, half of the centers (50%) reduce these levels before switching on the microphone. Just after switching to live mode, almost all centers (93%) increase or decrease the maximum levels based on the recipient's perception and some (45%) also shift the minimum levels. A small number of centers perform some kind of psychoacoustic test immediately after switch-on, for example, filtered Ling sounds loudness scaling (Nijmegen), Ling sounds detection (Perth), or closed or open set word understanding (Paris, Chapel Hill).

Most centers (76%) send the CI user home with incremental MAPS after the switch-on session. These MAPS contain progressively higher maximum levels allowing the CI user to accommodate to each MAP before switching to the next one. Some centers (17%) set a large volume range and instruct the CI recipient to increase the volume progressively over time. One center (Hannover) replied that they do not systematically increase the maximum level over time.

#### 3.2.4. Other MAP Parameters

**Figure 1:**
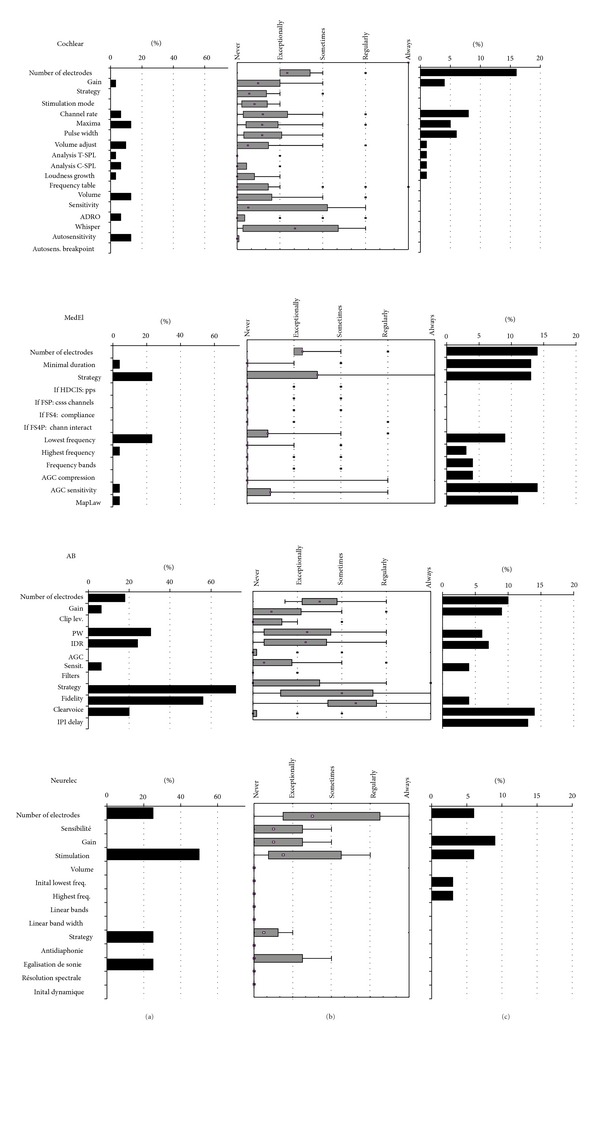
Occurrence of MAP changes for the 4 brands (Cochlear, Med-El, AB, and Neurelec). (a) The left panel shows the frequency of changing the default settings at switch-on, as retrieved from the questionnaire and the interview; (b) the mid panel shows the distribution of the frequencies of changing the MAP parameters during the followup sessions, as retrieved from the questionnaire and the interview (Box and Whisker plots with the central dot depicting the median value, the box shows the quartile range and the whiskers show the range); (c) the right panel shows the occurrence of MAP changes as observed in the cross sectional snapshot.

Figure 1(a) shows that other MAP parameters are rarely modified from default during the switch-on session. 

*Cochlear*. Thirteen percent of centers prefer more than the default 8 Maxima (9, 12 or 14) and 6% combine this with a higher than default Channel Rate (1200 pps). The Autosensitivity function is switched off by 13% of centers at switch-on. The Eargroup in Antwerp sets different Gains (statistically defined profile), and Analysis T-SPL (20), Analysis C-SPL (70) and switches off the ADRO function. The latter is also done by Nottingham where T-SPL is set to 25 dB and C-SPL is set to 75 dB at switch-on, in combination with a Q-factor of 16; both ADRO and ASC are deactivated. Paris also sets the Loudness Growth Function (Q-factor) at 16. The Volume Adjustment is set to 0 by 10% of centers, all located in the UK. 

*Med-El*. With the Med-El device, 23% of centers start with a different strategy than the default FS4 strategy. Chapel Hill provides the patients with two strategies, HDCIS or FSP, which are the two strategies approved for use in the USA. Perth lets the patients chose between FS4 and FS4p and has experienced that 90% of recipients prefer FS4p. Paris-Avicenne gives FS4p as startup strategy and York, Paris-Beaujon, and Kansas City give FSP as start-up strategy. The lowest filter frequency is set to 70 Hz by 23% of the centers. Paris-Avicenne overrules the default settings for Highest Frequency (set to 8000 Hz), AGC Sensitivity (set to 85%), and MapLaw (set to 1000), Nottingham overrules the default Minimum Pulse Width Duration (set to 20 *μ*s), and Nijmegen uses a high MapLaw setting (1000). 

*Advanced Bionics*. With the AB device a majority of centers overrule the default strategy (HiRes-P) and start with the HiRes-S strategy (72%), and of those, two-thirds select the Fidelity 120 strategy compared to one-third who stay with the default setting with Fidelity 120 switched off. This is in contrast to the centers who keep the HiRes-P strategy, of which 78% also keep the default setting with Fidelity 120 off. Some centers (20%) switch on Clearvoice systematically and some centers (30%) change the default Pulse Width setting of 10.8 *μ*sec to either a higher value or to the automatic Pulse Width algorithm II (APW2). The default input dynamic range (IDR = 60 dB) is changed by 24% of centers. Some lower it to 50 dB (*Las Palmas*, Paris-Avicenne) or 54 dB (Naples) while others increase it to 70 dB (London St-Thomas, Beirut, and Kerala) or to 80 dB (Antwerp). Antwerp also sets the sensitivity to −10 dB and the Gains to a preset profile which differs from the default values (0 dB). 

*Neurelec*. The statistics of Neurelec's Digisonic device are not solid since they are derived from merely four centers, one of which (Southampton) only uses the binaural version. Half of them change the default number of maxima from 12 to 11 (Antwerp) or 6, depending on the duration of deafness (Southampton), and one center (Antwerp) switches the stimulation rate systematically from 600 pps to 500 pps and the preemphasis (égalisation de sonie) to −1.

#### 3.2.5. Time

**Figure 2:**
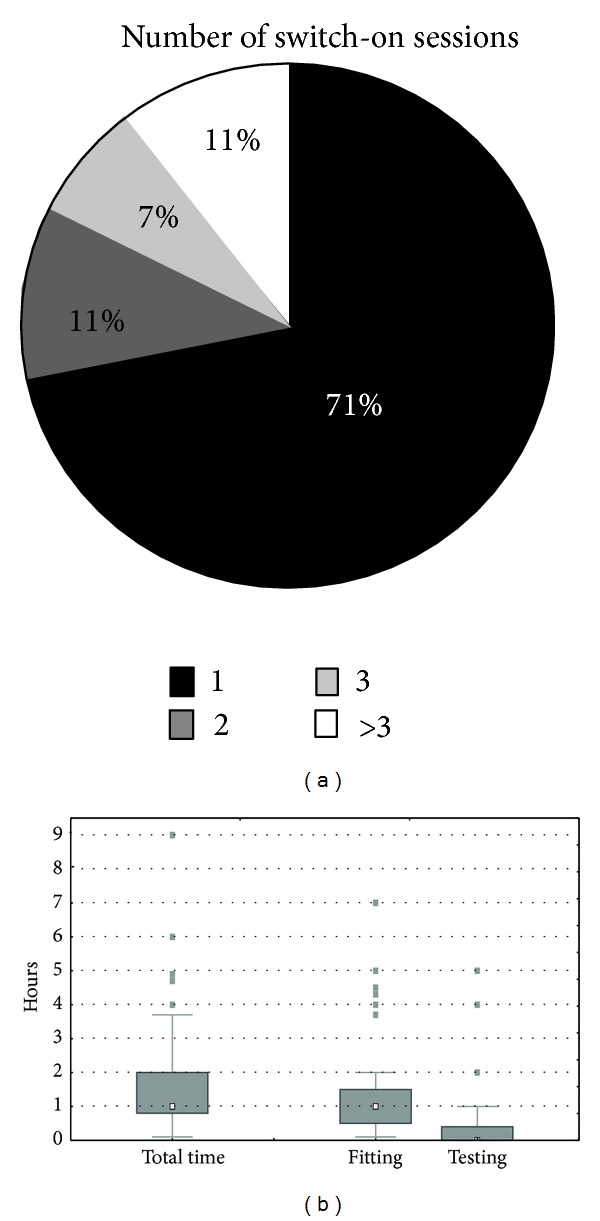
Time analysis of the switch-on session, showing the number of sessions at daily intervals which are considered to constitute the switch-on procedure (pie chart at the left) and the time spent at the switch-on session (box and whisker plot at the right), both the total time and its breakdown into time spent at fitting and at testing. Time for counseling has not been enquired in this study. See caption of Figure 1 to interpret the box and whisker plots.

Figure 2 shows that 71% of the centers consider the switch-on as 1 single session. Eleven percent spread the switch-on over 4 sessions or more, often on consecutive days. One center organizes the switch-on over 7 sessions (Oslo). The median cumulative time spent is 1 hour. This does not take into consideration the time spent for counseling or instructing the patient. As said earlier, testing is rarely undertaken during the switch-on session. There are outliers who spend more than 3 hours on fitting (Coimbra, London St-Thomas, Freiburg, Southampton, Kiel, and Oslo) or more than 1 hour at testing (Oslo, Montpellier, and Kiel). One center reports spending no more than 5 minutes in total, which is the result of a fully automated switch-on (Antwerp). There are no statistically significant differences between centers who perform cochlear implantations mainly in children compared to mainly in adults (Mann-Whitney *U* test *P* > 0.05).

### 3.3. Followup Procedures

After the switch-on session, all centers schedule a number of consecutive sessions to reach stable MAP settings. The average center schedules 3 sessions in the first quarter, 3 sessions in the following 3 quarters, and 1 annual session thereafter (see further). Attention goes mostly to the verification and adjustments of minimum and/or maximum levels to optimize loudness and almost half of the centers (46%) explicitly say that the followup sessions are roughly the same as the switch-on session.

#### 3.3.1. Adjustment of Minimum and Maximum Levels

All centers adjust maximum levels and many of them (61%) also adjust minimum levels. Global shifting of the maximum profile is very common (96%) while tilting is done by less than half of the centers (39%). One centre lets the CI-user set and balance his/her own maximum level to most comfortable (Grenoble). All centers perform some kind of loudness balancing across individual electrodes and some centers perform pitch ranking (17%).

Psychoacoustical tests (tonal audiometry, speech audiometry) or objective measures (eCAP, ESRT) are commonly performed (see below). Fourteen percent of the centers report using these early stage sessions to try out different strategies or different settings of MAP parameters other than minimum and maximum levels.

#### 3.3.2. Adjusting Other MAP Parameters

**Figure 3:**
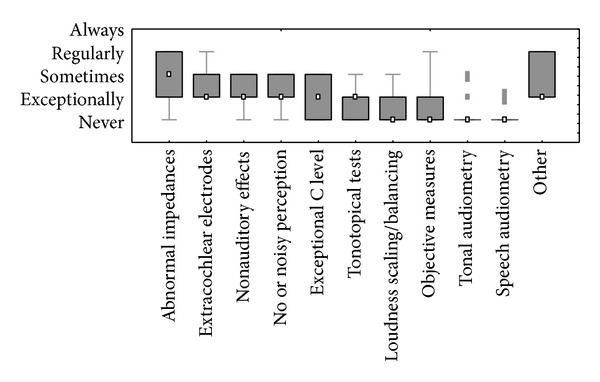
Alleged reasons for deactivating electrodes and the frequency they are reported to be really responsible for electrode deactivation in daily live.

Figure 1(b) shows that MAP parameters other than minimum and maximum levels are rarely modified. This is further illustrated by Figure 1(c) showing the cross-sectional observations. Deactivation of electrodes is one of the more common actions, but centers still report to doing this only every now and then (median response value is between exceptionally and sometimes, corresponding to approximately 10–15% in the cross-sectional data).  Figure 3 shows the reasons reported to deactivate electrodes. The most commonly reported reason is abnormal impedances, which is reported to occur “sometimes”. Electrodes are also deactivated for other reasons such as when there is an indication of extracochlear location, if they cause nonauditory stimulation, uncomfortable perception or if they are inaudible, if the maximum levels are exceptionally high, or if tonotopical tests such as pitch ranking, channel separation, or spectral discrimination show unexpected results. These situations are reported to occur almost never. Electrodes are hardly ever deactivated based on loudness assessment or objective measures. Other exceptional reasons of electrode deactivation are negative results on an integrity tests or the desire to increase the stimulation rate. One centre used to systematically start with one or more deactivated electrodes (Leiden) [9], a practice which has only recently been abandoned.

*Cochlear*. With the Cochlear device, the additional MAP parameter which is modified most, though still only exceptionally, is the Autosensitivity feature, which is then deactivated. In the cross-sectional data, also channel rate, number of maxima, and pulse width were modified in 5–8% of cases. 

*Med-El*. With the Med-El device, the strategy is reported to be changed in “some” cases. Some centers change the default strategy (FS4 except in the USA) to FSP or FS4p in exceptional or some cases. One centre routinely sets the strategy to HDCIS in the primary program (Chapel Hill) and lets the patient choose between this strategy and FSP. This was confirmed in the cross-sectional data, which also showed that AGC Sensitivity, Minimum Duration, and MapLaw were changed in 11–14% of the cases and by many centers (36–79%).

*Advanced Bionics*. Advanced Bionics has more MAP parameters modified by a substantial number of centers in the course of the early followup period. The Clearvoice feature is activated sometimes to regularly (14% of cases in the cross-sectional study and 36% of the centers), and also the Fidelity 120 feature is sometimes changed. Pulse width and IDR are next in line, but they are only changed in exceptional cases. This is confirmed by the cross-sectional data where these MAP parameters were only changed in 4–7% of the cases and by less than 25% of the centers. In the cross-sectional data the pulse rate was more often changed (IPI delay, 13% of cases, and 29% of centers). 

*Neurelec*. Neurelec again has too few data to allow any reliable statements. The results are nevertheless included in the graphs for completeness. 

#### 3.3.3. Time

**Figure 4:**
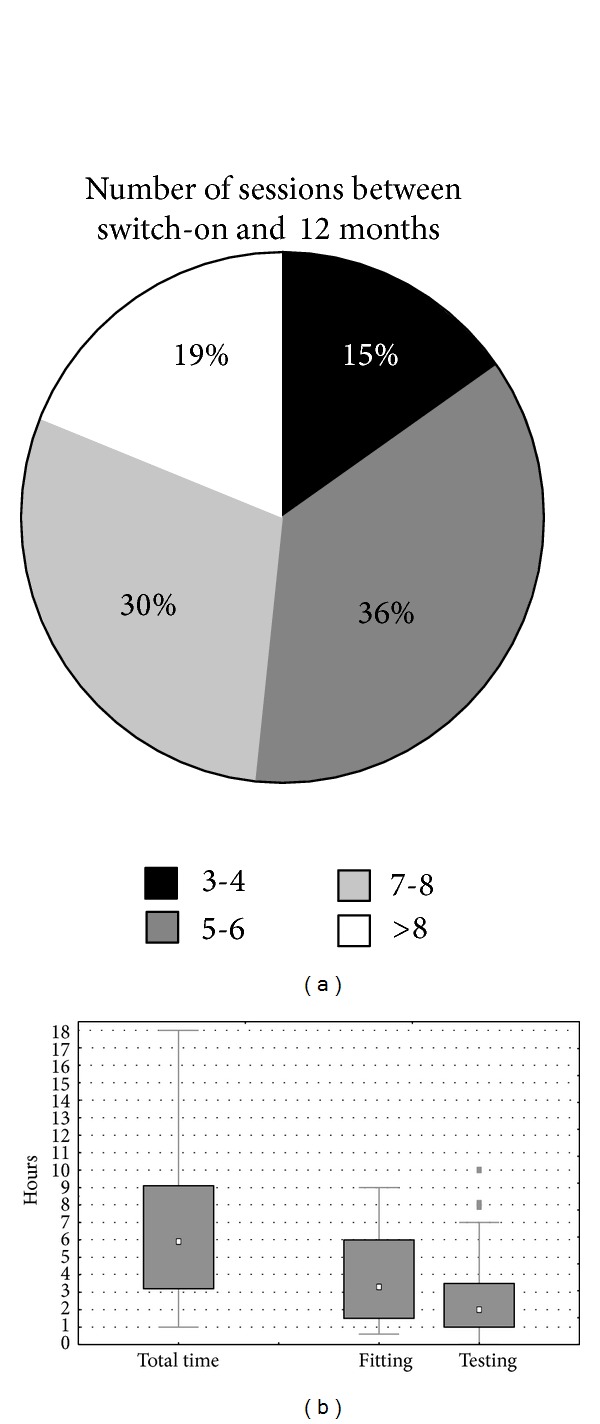
Time analysis of the follow-on sessions during the first year after switch-on, showing the number of sessions (pie chart at the left) and the cumulative time spent at them (box and whisker plots at the right), both the total time and its breakdown into time spent at fitting and at testing. Time for counseling has not been enquired in this study. See caption of Figure 1 to interpret the box and whisker plots.

Figure 4 shows that most centers schedule between 5 and 8 additional sessions during the first year (median = 6; QR: 5–8, range: 3–15). The median cumulative time spent at the acts of fitting and testing during the first year after switch-on is 6 hours (median for fitting = 3.3 hours and for testing = 2.0 hours). There are no significant differences between centers who perform cochlear implantations mainly in children compared to mainly in adults (Mann-Whitney *U* test *P* > 0.05).

After the first year, the median number of sessions per year is 1 (QR: 1-1, range 0.3–1 with one outlier with 3 annual sessions). The median time spent is 1.3 hours (QR: 0.9–2.0 hours, range 0.5–4 hours with one outlier of 8 hours per year) of which 0.5 hour for fitting and 0.8 hour for testing.

### 3.4. Outcome Measurements

**Figure 5:**
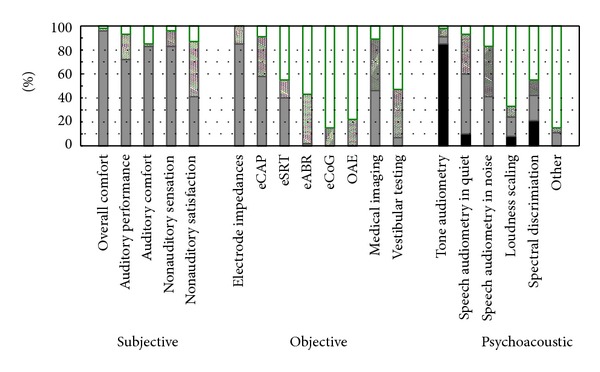
It shows the different outcome assessments which were enquired in the questionnaire together with the frequencies of the responses. The outcomes are grouped into 3 groups (subjective, objective, and psychoacoustic outcomes). The possible answers were (1) yes we assess this and use it to optimize the fitting (solid black and grey bars), (2) yes we assess this but for other reasons than steering the fitting, like for documentation or longitudinal followup (shaded bars), or (3) no we do not use to assess this (white bars). For the solid bars (assess and use it) a distinction was made into whether they have well defined targets to reach (black) or not (grey).

Figure 5 shows that most centers report assessing subjective features and using them for fitting. Overall comfort (93%), auditory comfort (83%), and the presence of nonauditory sensations (83%) are used by most centers. None of the centers reports well defined and measurable targets for any of these features. Non auditory satisfaction, such as contentment, quality of life, implant use, are commonly assessed (87%) but only used by 41% of the centers to change the MAP settings.

Of the objective measures [10], electrode impedances are measured by 100% and used by 85% of the centers. They are used to deactivate electrodes in case of short or open circuit. Thresholds based on eCAP [11] or eSRT [12] measurements are used by 59% and 39% of the centers, respectively. They are mainly used to set the MAP profiles. Medical imaging is used by 46% of the centers to change the fitting, mainly to deactivate electrodes which are believed to be extracochlear. Other objective measures may be performed, but they are not used to drive the fitting. None of the centers report using objective measures to reach well defined targets during the fitting, except for Nottingham, where ESRT measures are used for loudness balancing of the MAPs. The cross-sectional data confirmed that, besides impedance measurements, no objective measure was performed in more than 5% of the cases.

**Figure 6:**
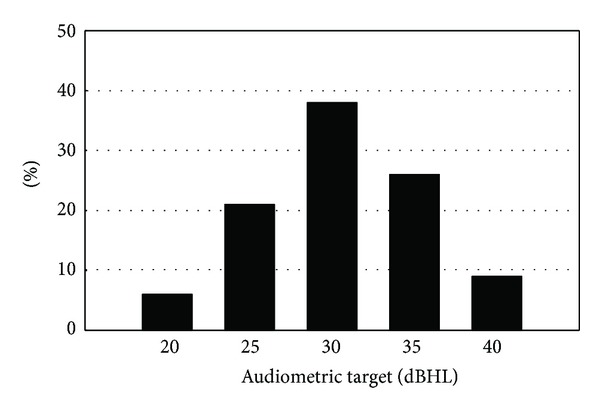
Histogram showing the frequency of the reported audiometric targets (dBHL) at different centers.

Psychoacoustic measures are the only outcome measures for which a number of centers have well defined targets. This holds mainly for pure tone audiometry (85%) with targets set between 20 and 40 dBHL (median 30 dBHL, QR: 25–35 dBHL; see Figure 6). Spectral discrimination tests are used to drive the fitting by 41% of the centers of which 20% use well defined targets (either 100% if the A§E phoneme discrimination test [13] is used or 83–100% if Ling sounds are used). Speech audiometry in quiet or in noise are reported to be used to change the MAP parameters by 61% and 41%, respectively, but only 11% of the centers have set well defined targets and this is only for speech audiometry in quiet. No two centers have set the same target for this measure; however, acoustical loudness scaling is used to change the fitting by 24%, but only 8% have well defined targets, which are the same across centers, namely, results falling in the normal zone (of hearing listeners).

The cross-sectional data confirm that free field audiometry was performed in 60% of the cases, speech audiometry was performed in quiet in 45%of cases, speech audiometry was performed in noise in 19% of cases, loudness scaling was performed in 11% of cases, and spectral discrimination tests were performed in 15% of the cases. Other tests used were speech tracking and Ling sounds detection, discrimination and loudness scaling tests, but these were very rare.

## 4. Discussion

Multichannel intracochlear implants have been clinically available for more than 25 years. The fitting of the processors to the individual recipient is considered to be crucial in obtaining good results. To date there is neither well described and commonly adopted Good Clinical Practice (GCP) for this act nor evidence based material to distinguish efficient procedures from less efficient ones. Over these 25 years, fitting a CI has been carried out by competent clinicians who have established their own heuristics, good practices, and empirical knowledge. It seems reasonable to believe that a critical analysis of the cumulative knowledge acquired over the years may serve as a first step towards a definition of GCP. This report attempts to give an inventory of the current state of the art as it is based on a vast number of CI centers worldwide. All together they represent over 47000 CI recipients and 93% of the participating centers have more than 10 years of experience. 65% of the centers are European, which may cause a bias towards an overrepresentation of European habits. Altogether this is an unprecedented inventory and we believe that it gives a representative view on the current practices in CI fitting, which may be considered as the benchmark of CI fitting in 2013.

It is important to consider that the conclusions are based on a compilation of a written questionnaire, an oral interview, and a cross-sectional fitting data snapshot. An intrinsic weakness of such an approach is that it lacks precision. It is based on the anamnestic summary of centers' practices as provided by only one representative per center, whereas different practices may exist within one center. Yet, in the absence of hard evidence or more accurate overall data, an exploratory inventory like this one is a legitimate and necessary first step towards a better understanding of the field. The cross-sectional sample serves as verification and substantially improves the validity of the data. We advise the reader not to interpret the presented numerical data as ultimately accurate but rather as indicative while always keeping a confidence interval in mind.

A first observation is that most centers now offer 3 CI brands and perform cochlear implantation in both children and adults. This is different from years ago when many CI centers only offered one brand and only performed CI in adults.

A second observation is that, despite the huge variability across centers (see further), some common practices can be extracted and they would seem to be as follows.

The typical switch-on procedure takes one session comprising counseling and 1 hour of fitting. Testing is not performed at this stage. The fitting procedure is as follows:

1. connect the processor 4 weeks after surgery;
2. measure impedances and deactivate electrodes in case of short or open circuits;
3. measure the maximum level behaviorally on a number of electrodes along the electrode array, and interpolate the others;
4. set the minimum level at 0 for Med-El, 10% of M for AB; for the other implants measure the minimum level behaviorally on a few electrodes and interpolate the others;
5. perform loudness balancing by presenting a signal on all electrodes successively;
6. reduce the maximum level and switch on the microphone;
7. let the CI recipient accommodate for a few minutes and ask whether sounds, including loud sounds, are tolerated; increase or decrease the entire profile of maxima in order to make loudness tolerable or comfortable;
8. put a number of progressive MAPs in the processor;
9. instruct the patient to accommodate to each program for a couple of days and switch to the next one afterwards.

The typical first-year followup would comprise three monthly sessions followed by three quarterly sessions of one hour each. The sessions would typically look like this:

1. perform pure tone audiometry and speech audiometry (in quiet);
2. measure impedances and deactivate electrodes in case of short or open circuits;
3. verify the levels on individual electrodes by loudness balancing;
4. shift the profiles of the maximum and, if necessary, also of the minimum levels globally;
5. if deemed necessary, tilt the maximum levels globally;
6. define own criteria to identify selected and exceptional cases in whom other MAP parameters are modified.

We believe that the description of these typical procedures is the common denominator of current practices and could serve as guidelines for newcomers in the field, as the backbone of instructional courses, and so forth.

But, as said, it is remarkable to observe the substantial variability across centers and this holds for virtually all aspects of CI fitting and followup. Each CI center has its own policy in terms of timing, content, and methodology.

The switch-on of the processor is scheduled between 2 and 6 weeks after surgery. It is obvious that concern about wound healing is a reason not to activate the processor too early. On the other hand one does not like to deprive the CI recipient from audition for too long a period and it has been shown that the natural and progressive increase in electrode impedances after surgery is discontinued by electrical stimulation [5, 14]. These may be factors in favor of early device switch-on. The observation that some centers commonly activate the processor as soon as 2 weeks after surgery seems to suggest that 2 weeks may still be well within the safe time window.

On average, CI recipients undergo one switch-on session followed by six fitting sessions in the first year, each taking approximately one hour of technical interaction (fitting and testing) plus a considerable amount of counseling time which has not been enquired in this survey. Behind this average there are huge between-centers differences. Even within one center there may be important differences between different clinicians and between different patients (e.g., children compared to adults). Most centers have one switch-on session followed by a take-home experience for accommodation. Some centers however schedule 5 to 7 consecutive sessions at daily intervals (Hannover, Freiburg, and Oslo). This may be based on the experience that such intensive schemes lead to stable MAPS fast or it may be for practical reasons, for instance, for patients who are living at a distance from the CI center. In some cases this is compensated by fewer followup sessions in the first year, as in Hannover and Oslo, where no more than 4 followup sessions are scheduled in the year after switch-on, but not in Freiburg, where 10 more sessions are planned in the first year, a scheme which fits in a well established rehabilitation concept. In the year following switch-on, some centers spend no more than approximately 1.5 to 2.5 hours (Paris-Avicenne, *Casablanca*, Ghent, Pune, Mumbai, Hannover, Berlin, Valencia, and Lyon) and one center schedules only 3 sessions (Warsaw), while other centers spend at least 12 hours (*Las Palmas*, Leiden, London St-Thomas, and Amsterdam) or as much as 15 sessions (Brussels). After the first year, there is more consistency in terms of followup. Almost all centers have one session per year which takes between 1 and 2 hours of technical interaction (fitting and testing) with the CI user. Three centers have less than 1 annual session (Hannover and London-RNTNE every second year, Nijmegen every third year, and Mumbai on patient's request). It seems that these annual sessions are merely planned for verification and to reassure that the performance has not deteriorated, rather than for modifying the MAPs which remain rather stable after the initial months [15]. From that perspective it seems justified to increase the interval of one year. However, informal feedback from centers has revealed that these annual visits are also felt to be important for technical checkup of the processor and the microphone function and for ongoing counseling. When asked about this during the above mentioned international debate, 62% of the participants voted that annual visits were essential during the first 5 years and this figure dropped to 28% after 5 years.

When it comes to the content of fitting and followup, most attention goes to the setting of minimum and maximum levels per electrode. Every center appears to have its own policy on how to determine these levels. Behavioral assessment is commonly used, but, whereas this was performed for each individual electrode in the past, it now seems common to assess the levels on a few electrodes only and to deduce them by interpolation for the remaining electrodes. This probably coincides with the change from bipolar to monopolar stimulation and is based on growing evidence that such approach yields equally good results [16]. Evoked potentials (mainly eCAP thresholds) are used by an important minority of the centers, but they appear to be used as global indication of minimum or maximum levels rather than as strict anchor points. The levels set this way are preliminary anyhow, since they are shifted and to a lesser extent tilted in live mode, mainly based on subjective appreciation of loudness [17]. Whereas many reports correlate eCAP based MAPs with behaviorally based MAPs, as far as we know there are no reports claiming to improve speech understanding when eCAP based MAP optimization is carried out. On the contrary, Smoorenburg concluded that the applicability of eCAP measures in processor adjustment could not be demonstrated [18]. Other MAP parameters are rarely modified. Some default settings are systematically overruled by a large number of centers, which probably reflect their conviction that the default settings do not necessarily give the best results. It seems obvious for CI companies to take this into consideration and to change some of their default settings. Deactivating electrodes is the most frequent next MAP modification, although this remains rare. This may be subject for reflection since indication exists that selectively deactivating electrodes may substantially improve auditory performance. When asked whether the selective dropping of one electrode may cause a significant improvement on speech understanding, 95% of the participants in the debate voted affirmatively. However, it remains difficult to identify such electrodes. The current survey demonstrates that centers have their own and often different methods to do so (Figure 3). Finding a valid method to identify electrodes, which, when deactivated, cause a significant improvement in auditory performance, may be a very legitimate subject for future research. It remains puzzling whether modifying the many other MAP parameters is relevant or not. One of the problems encountered is the difficulty to correctly understand the function of these parameters and to predict the effect of modifying them. We have developed a uniform graphical representation for all four commercially available implant systems to clarify this behavior in the acoustical, electronic, and electrical domain [19] and we hope that the interactive application which has been developed to simulate the devices' behavior will be instrumental for clinical use. To the best of our knowledge, no scientific studies exist to explore a systematic impact of modifying parameters like the Input Dynamic Range, the Sensitivity, the AGC Compression factor, the MapLaw, and so forth. Hence, discussing the relevance of this can only be subject to speculation.

The most striking is the observation that the centers rely mostly on the recipient's subjective feedback to drive the MAP changes. This is remarkable since many CI users have no clear reference point to estimate the subjective quality of sound, either because they have never had normal hearing before or because they have been deprived of normal hearing for many years and have got used to hearing aid sound over the years. Also such subjective feedback is not quantitative and can therefore hardly lead to systematic process optimization, since this fundamentally requires measured results and targets. In addition many experienced clinicians indicated that patient's subjective judgment may not coincide with optimal performance. Objective measures are only used to get a prior estimate of the shape of the minimum or maximum levels, but they almost never serve the fine tuning of the device.

The expectation might be that, after more than 25 years of cochlear implantation, the field had developed psychoacoustic targets to steer the device fitting, but this survey shows that targets only exist in terms of audiometric thresholds. One of the reasons for this may be the fact that it is not always obvious how to modify the MAP parameters if targets are not met. Current CI systems are complex and predicting their behavior after changing the MAP is not obvious. On the other hand, such reasoning is also vicious and defining targets may be an incentive to explore and develop procedures to achieve target in the most efficient ways. Most centers agree on a target of 30 dBHL (±5 dB) for most audiometric frequencies, and this is achievable with current microphones and front-end processing.

Auditory performance, however, hardly depends on thresholds but rather on supraliminal sound processing. The core function of the cochlea is discriminating the different features of sound, such as loudness, spectral content, and temporal content, and it is striking to see that less than 50% of the centers report basing their fitting on measures to assess this and that less than 25% report having targets in this domain [13, 19]. Speech audiometry in quiet or in noise relates to the daily auditory performance but depends on more than just the cochlear processing of sound. Therefore speech audiometry is only partly indicative of the quality of cochlear functioning. Speech audiometry is used by approximately half of the centers but most use it to monitor performance, that is, to detect any undesired deterioration over time. Only 11% report having well defined speech audiometrical targets when it comes to CI fitting. This is in line with instructional literature which extensively explains the available methodology and how to use it to determine the minimum and maximum levels but which avoids mentioning measurable targets [2, 6, 20–22]. Shapiro coined the term “common lethargy” when referring to the CI audiologists' willingness to consider changes in device programming and he correctly stated that device programming is not a goal per se but the absolute goal is to provide the patient with a comfortable program which ensures maximum performance [2].

In conclusion, it seems fair to summarize the current state of CI fitting as setting global profiles of maximum current levels and to a lesser extent of minimum current levels, mainly based on subjective feedback from the CI user. Many different approaches exist and in the absence of targets or well defined outcome measures it seems impossible to compare all these differences and to judge whether some yield better results or are more efficient than others. It is likely that several approaches in the hands of different experts may lead to similarly good results. It is equally likely that defining common measurable targets may be a next step to be taken towards the optimization of the art of fitting.

## Supplementary Material

This questionnaire was distributed to and returned by 47 CI centers worldwide. It served as baseline for the international debate (Antwerp, 22-23 October 2012) and the subsequent individual interviews for verification.
Briefly the questions focused on the following topics:
1. Number of implant recipients being followed and the annual increase2. Brands of implants being implanted and fitted3. MAP parameters being modified from default at switch-on and during the followup4. Assessments undertaken (subjective, objective, and psychoacoustic) and used to steer the MAP modifications5. Well defined targets used
