# Supplementary material for: Cochlear Implant Programming: A Global Survey on the State of the Art
Source: ScientificWorldJournal. 2014 Feb 4;2014:501738. doi: 10.1155/2014/501738 (PMC3932199; doi:10.1155/2014/501738)
Supplement: Supplementary file 1 — This questionnaire was distributed to and returned by 47 CI centers worldwide. It served as baseline for the international debate (Antwerp, 22-23 October 2012) and the subsequent individual interviews for verification. Briefly the questions focused on the following topics: 1. Number of implant recipients being followed and the annual increase2. Brands of implants being implanted and fitted3. MAP parameters being modified from default at switch-on and during the followup4. Assessments undertaken (subjective, objective, and psychoacoustic) and used to steer the MAP modifications5. Well defined targets used [file 501738.f1.pdf]

COCHLEAR IMPLANT FITTING: CURRENT PRACTICE

Together we can improve it

Thank you for taking the time to fill out this survey. It takes no longer than 10 minutes for the first 8 pages and somewhat more time if you are so kind as to also fill out the optional pages at the end.

The aim of this survey is to get a better insight into the current practice of cochlear implant fitting. We have selected you because we believe you are involved in the fitting of cochlear implants.

You can choose to fill out the forms directly on our site
http://www.otoconsult.com/feedback/FittingSurvey.aspx

If on paper, please return this survey to debate@otoconsult.com or to

Otoconsult
Drs Bart Vaerenberg
Herentalsebaan 71
B-2100 Antwerp
Belgium

We will use the following definitions:

Service : a relatively autonomous group of professionals with its own head or director, its own staff and its own budget. E.g. an ENT department within a hospital could be a service on its own. If the clinical audiology is organized within this ENT department, it is part of the same service.

In-house : located in the same building or on the same campus.

Medical : relating to all non-surgical medical activities, such as otoscopy, diagnostics, prescription of medication, pediatric care, etc.

Surgical : relating to the surgical intervention of cochlear implantation

Audiological : relating to clinical audiology, e.g. audiometry, speech audiometry, evoked potentials (ABR), etc.

CI Fitting : the act of programming the CI processor by means of the CI fitting software.

Rehabilitation : relating to speech therapy, auditory rehab, psycho-social counseling, family guidance, etc.

Audiologist : a professional, other than a medical doctor, with a non-university or university training in audiology, spending most of his/her time at clinical audiology, hearing aid fitting or auditory rehabilitation.

1. Personnel

Please indicate the number of full time equivalent persons contributing to your service for each of the following categories.

We are interested in getting a better view on the structure of your organization. Your service has its own head who autonomously decides on the budget and the personnel. The question relates to this your service.

CI Surgeons	
Medical Doctors (non-CI surgeons)	
Audiologists	
Speech Therapists	
Administrative	
Other (please specify)	

2. Contact Information

Please tell us who you are. We will treat this information as confidential and will not share it with third parties. We will make a report of this survey available to you asap.

Clinic / Centre	
Address	
Address 2	
Postal Code	
City	
Country	
Email	
Website	

3. Activities

Which of the following CI related activities are performed and where?

The organization of cochlear implantation requires a multi-disciplinary approach. We have identified a number of activities that are typically involved. They may be performed by your own service , by another service in-house or by an external service. Could please indicate how this is organized?

	Own service	Other service, in-house	External service	Not performed
Medical (ENT, pediatrics, etc.)	[ ]	[ ]	[ ]	[ ]
Surgical (cochlear implantation)	[ ]	[ ]	[ ]	[ ]
Audiological (clinical audiology)	[ ]	[ ]	[ ]	[ ]
Cochlear implant fitting (programming)	[ ]	[ ]	[ ]	[ ]
Rehabilitation	[ ]	[ ]	[ ]	[ ]
Other	[ ]	[ ]	[ ]	[ ]

Other Activities

If you've added other activities in the previous question, please specify them here.

Description	

5. First Implantation

In which year did your service first fit a cochlear implant?

Year:	

6. Total number of implantations

Can you give a rough estimate of the total number of implants that have been switched on in your service (since your service started doing cochlear implantation)?

less than 100	
100-300	
300-1000	
1000-5000	
more than 5000	

7. Number of implantations per year

Can you give a rough estimate of the annual number of implants that have been switched on in your service (e.g. last year)?

less than 10	
10-30	
30-100	
more than 100	

8. Adult / child ratio

Can you give a rough estimate of adult/child ratio during the last 3 years?

only adults	
more adults than children	
roughly equal numbers	
more children than adults	
only children	

9. If you use Cochlear: do you use Customsound's default settings for initial stimulation?

Customsound comes with default settings for a number of parameters. Can you indicate whether you usually accept them or whether you change them before you switch on the processor for the first time?

	I change Custom-Sound's default value	The value I usually set before initial stimulation	Explain why you prefer your value over the Customsound default value
C (default = 102 CU on all electrodes)	[ ]		
T (default = 100 CU on all electrodes)	[ ]		
Gains (default set to 0)	[ ]		
Strategy (default = ACE)	[ ]		
Stimulation Mode (default = MP1+2)	[ ]		
Channel Rate (default = 900 Hz)	[ ]		
Maxima (default = 8)	[ ]		
Pulse Width (default = 25us)	[ ]		
Volume Adjustment (default = 20% DR)	[ ]		
Analysis T-SPL (default = 25)	[ ]		
Analysis C-SPL (default = 65)	[ ]		
Loudness Growth (default = 20)	[ ]		
Frequency table (default = 22 (188-7938))	[ ]		
Power (default = Auto)	[ ]		
Volume & Sensitivity	[ ]		

9. If you use Med-El: do you use Maestro System Software's default settings for initial stimulation?

Maestro System Software comes with default settings for a number of parameters. Can you indicate whether you usually accept them or whether you change them before you switch on the processor for the first time?

	I change the default value	The value I usually set before initial stimulation	Explain why you prefer your value over the Maestro System Software default value
Number of active electrodes (default = 12)	[ ]		
MCL Charge (default = 0 qu on all electrodes)	[ ]		
THR Charge (default = 0 qu on all electrodes)	[ ]		
Min. Dur. (default = 7,08 us)	[ ]		
Strategies (default = FS4)	[ ]		
if HDCIS: pps (default = max=4225)	[ ]		
if FSP: csss channels (default = max=5)	[ ]		
if FS4: compliance (default = autoadjust)	[ ]		
if FS4-P: channel interaction compensation (default = 3 for apical and 3 for basal)	[ ]		
Lowest frequency "from ..." (default = 100 Hz)	[ ]		
Highest frequency "to ..." (default = 8500 Hz)	[ ]		
Frequency bands (default = Logarithmic FS)	[ ]		
AGC Compression Ratio (default = 3:1)	[ ]		
AGC Default Sensitivity (default = 75%)	[ ]		
MapLaw (default = Logarithmic)	[ ]		

9. If you use Advanced Bionics: do you use SoundWave's default settings for initial stimulation?

SoundWave comes with default settings for a number of parameters. Can you indicate whether you usually accept them or whether you change them before you switch on the processor for the first time?

	I change Sound-Wave's default value	The value I usually set before initial stimulation	Explain why you prefer your value over the SoundWave default value
M (defaults to 0 CU on all electrodes)	[ ]		
T (default is Set all T's to 1/10 of M)	[ ]		
Gains (default set to 0)	[ ]		
Electrode status (by default all electrodes are enabled)	[ ]		
Clipping Level (Clipping is disabled by default on all electrodes)	[ ]		
Pulse Width (default setting is "Automatic 1" (10.8 µs))	[ ]		
IDR (default is 60dB)	[ ]		
AGC (enabled by default)	[ ]		
Sensitivity (0dB by default)	[ ]		
Filters ("Extended Low" by default)	[ ]		
Volume Range Upper Limit (Default 20%)	[ ]		
Volume Range Lower Limit (Default 50%)	[ ]		
Audio Mixing (50/50 Mic/Aux by default)	[ ]		
Strategy (default is HiRes-P)	[ ]		
Fidelity120 (disabled by default)	[ ]		

9. If you use Neurelec: do you use Digimap's default settings for initial stimulation of Saphyr processors?

Digimap comes with default settings for a number of parameters. Can you indicate whether you usually accept them or whether you change them before you switch on the processor for the first time?

	I change Digimap's default value	The value I usually set before initial stimulation	Explain why you prefer your value over the Digimap default value
Active Electrodes (default = 20)	[ ]		
Min (default = 5 on all electrodes)	[ ]		
Max (default = 5 on all electrodes)	[ ]		
Sensibilité (Sensitivity) (default = 0)	[ ]		
Gain (default = 0)	[ ]		
Stimulation (default = 12)	[ ]		
Volume (default = 0)	[ ]		
Intenstié (amplitude) (default = 70)	[ ]		
Puissance d'émission (transmission power) (default = 15)	[ ]		
Lowest frequency (default = 195)	[ ]		
Highest frequency (default = 8008)	[ ]		
Linear bands upto... (default = 1367)	[ ]		
Linear bandwidth (default = 130)	[ ]		
Strategy (default = 600 pps)	[ ]		
Optimisation anti-diaphonie (default = off)	[ ]		
Egalisation de sonie (default = 0)	[ ]		
Résolution spectral (default = haute)	[ ]		
Dynamique (default = haute)	[ ]		

10. Subjective Patient Feedback

Please indicate how you evaluate the patient's subjective feedback and how you use this for fitting.

We are interested in when and how often you assess these subjective measures in a single patient (on what indication do you assess them), and whether they influence the fitting of the processor. If the feedback of the patient has impact on the fitting, please explain which processor parameters you adjust , and how you adjust them based on the patient's feedback.

If you have any rules of thumb to adjust fitting based on a patient's feedback, please explain them. We would also like to know what targets you aim at for each of these assessments. For example, a target may be to avoid facial stimulation at all time. A target for overall performance may be the ability to have conversations over the telephone.

	Do you assess this?	When / How often / Indication?	Do you use the result for adjusting the program?	What are your targets for this measurement and how does the result influence fitting (do you have rules of thumb)?
Subjective overall auditory comfort (sound quality, loudness, noisiness, etc.)	[ ]		[ ]	
Subjective overall auditory performance (speech understanding in daily situations, use of telephone, music appreciation, etc.)	[ ]		[ ]	
Subjective auditory comfort on specific electrodes (tone bursts, speech bursts)	[ ]		[ ]	
Subjective non-auditory sensation (e.g. facial stimulation)	[ ]		[ ]	
Subjective non-auditory satisfaction (contentment, quality of life, frequency of wearing the implant, etc.)	[ ]		[ ]	
Pitch ranking between electrodes	[ ]		[ ]	
Other (please specify in the "When / How often / Indication" field)	[ ]		[ ]	

Comments: ....

11. Psychoacoustical / Audiological Measurements

Please indicate which of the following measurements you perform and how you use them for fitting.

We are interested in when and how often they are performed in a single patient (on what indication do you perform them), and whether they influence the fitting of the processor. If the results of the measurement have impact on the fitting, please explain which processor parameters you adjust , and how you adjust them based on the result of the measurement. If you have any rules of thumb to adjust fitting based on a psychoacoustical test result, please explain them. We would also like to know what targets you aim at for each of these measurements. For example, a target for tone audiometry may be to avoid any audiometric threshold of 40dB HL or more. A target for speech audiometry may be an SRT of 65dB SPL.

	Do you assess this?	When / How often / Indication?	Do you use the result for adjusting the fitting?	What are your targets for this measurement and how does the result influence fitting (do you have rules of thumb)?
Tone Audiometry	[ ]		[ ]	
Speech audiometry in quiet	[ ]		[ ]	
Speech audiometry in noise	[ ]		[ ]	
Loudness scaling	[ ]		[ ]	
Phoneme Discrimination	[ ]		[ ]	
Other (please specify in the "When / How often / Indication" field)	[ ]		[ ]	

Comments: ....

12. Objective Measurements and Imaging

Please indicate which of the following objective measurements you perform.

We are interested in when and how often they are performed in a single patient (on what indication do you perform them), and whether they influence the fitting of the processor. If the results of the measurement have impact on the fitting, please explain which processor parameters you adjust, and how you adjust them based on the result of the measurement. If you have any rules of thumb to adjust fitting based on test results, please explain them. We would also like to know what targets you aim at for each of these measurements. For example, you may want to aim for all electrode impedances to be below a certain value, and disable all electrodes that do not meet this requirement.

	Do you assess this?	When / How often / Indication?	Do you use the result for adjusting the fitting?	What are your targets for this measurement and how does the result influence fitting (do you have rules of thumb)?
Electrode Impedance measurements	[ ]		[ ]	
Neural Response Imaging (NRI)	[ ]		[ ]	
Stapedial Reflex Thresholds (SRT)	[ ]		[ ]	
Auditory Brainstem Responses (ABR)	[ ]		[ ]	
Other Evoked Potentials (ECAP, WNAP, Middle Latency, ...)	[ ]		[ ]	
Electrocochleography (ECoG)	[ ]		[ ]	
Otoacoustic Emmissions (OAE)	[ ]		[ ]	
Medical Imaging (X-ray, CT, MRI, ...)	[ ]		[ ]	
Vestibular tests (Electronystagmography (ENG, VNG), Rotation tests)	[ ]		[ ]	
Other (please specify in the "When / How often / Indication" field)	[ ]		[ ]	

Comments: ....

13. Inter-fitter and inter-patient variabilities

Could you please estimate the contribution of the following factors to fitting variability?

Suppose you change one single factor in the fitting process, what do you think will happen to the programming of the processor? Will it be more or less the same or will it be very different?

	Exactly the same	More or less the same	Somewhat different	Quite different	Very different
If we change the audiologist performing the programming with another audiologist from within your own service, how will the resulting programs compare?					
If we change the audiologist performing the programming with another audiologist from another service, how will the resulting programs compare?					
If the same audiologist programs a child or an adult, how will the programs compare?					
If the same audiologist programs a prelingually deaf person or a postlingually deaf person, how will the programs compare?					
If the same audiologist programs an adult who has been deaf for less than a year or he/she programs an adult that has been deaf for more than 10 years, how will the programs compare?					

Comments: ....

14. Resources required on average for fitting and audiological measurements after implantation

Please indicate how much time is spent on the actual programming of the processor and how much is spent on audiological testing (this includes behavioral, objective and electrophysiological measurements)?

	Programming	Audiological Measurements
Device switch-on session		
Follow-up during 1st, 2nd and 3rd month after device switch-on		
Follow-up during 4th to 12th month after device switch-on		
Follow-up per year afterwards		

This last page is optional, though important.

15. Resources required to implant an adult

Please indicate how much time the following activities take on average and by whom they are performed?

We have identified a number of tasks that are involved in the preparation of an adult for implantation and in the follow-up after implantation. The question only relates to medical, surgical and audiological issues, not to rehabilitation nor family guidance at home etc.

	Medical Doctor / Surgeon	Audiologist	Speech therapist	Administrative worker	Other personnel
Medical and audiological assessment of CI candidates ("CI-selection")					
Adminsitrative preparation before surgery (e.g. telephone calls, reimbursement file, agenda planning, etc.)					
Surgery (including intra-operative testing)					
Follow-up between surgery and device switch-on					
Device switch-on session					
Follow-up during 1st month after device switch-on					
Follow-up during 2nd and 3rd month after device switch-on					
Follow-up during 4th, 5th and 6th month after device switch-on					
Follow-up during 7th to 12th month after device switch-on					
Follow-up per year afterwards					

Other Resources for adult implantation

If you've added other personnel in the previous question, please specify them here.

Description	

17. Resources required to implant a child

Please indicate how much time the following activities take on average for an individual implant recipient and by whom they are performed?

We have identified a number of tasks that are involved in the preparation of a child to the implantation and in the follow-up after implantation. The question only relates to medical, surgical and audiological issues, not to rehabilitation nor family guidance at home etc.

	Medical Doctor / Surgeon	Audiologist	Speech therapist	Administrative worker	Other personnel
Medical and audiological assessment of CI candidates ("CI-selection")					
Adminsitrative preparation before surgery (e.g. telephone calls, reimbursement file, agenda planning, etc.)					
Surgery (including intra-operative testing)					
Follow-up between surgery and device switch-on					
Device switch-on session					
Follow-up during 1st month after device switch-on					
Follow-up during 2nd and 3rd month after device switch-on					
Follow-up during 4th, 5th and 6th month after device switch-on					
Follow-up during 7th to 12th month after device switch-on					
Follow-up per year afterwards					

Other Resources for adult implantation

If you've added other personnel in the previous question, please specify them here.

Description	
